# Pathological Alterations in Human Blood Microbiome—An Updated Review

**DOI:** 10.3390/ijms26125807

**Published:** 2025-06-17

**Authors:** Stela Dragomanova, Reni Kalfin, Lyubka Tancheva, Sidharth Mehan, Dana Stanciu, Stefan Panaiotov

**Affiliations:** 1 Department of Pharmacology, Toxicology and Pharmacotherapy, Faculty of Pharmacy, Medical University of Varna, 84 A Tsar Osvoboditel Blvd., 9002 Varna, Bulgaria; stela_dragomanova@abv.bg; 2 Department of Biological Effects of Natural and Synthetic Substances, Institute of Neurobiology, Bulgarian Academy of Sciences, Acad. G. Bonchev Str. 23, 1113 Sofia, Bulgaria; lyubkatancheva@gmail.com; 3 Department of Healthcare, Faculty of Public Health, Healthcare and Sport, South-West University, 66 Ivan Mihailov St., 2700 Blagoevgrad, Bulgaria; 4 Division of Neuroscience, Department of Pharmacology, ISF College of Pharmacy, Moga 142001, Punjab, India; sidh.mehan@gmail.com; 5 Department of Pharmaceutical Botany, Faculty of Pharmacy, “Iuliu Hațieganu” University of Medicine and Pharmacy, 400337 Cluj-Napoca, Romania; dana.stanciu@elearn.umfcluj.ro; 6 National Centre of Infectious and Parasitic Diseases, Yanko Sakazov Blvd. 26, 1504 Sofia, Bulgaria; spanaiotov@yahoo.com

**Keywords:** blood, microbiome, nervous system, cardiovascular diseases, metabolite disorders, microorganisms, erythrocytes

## Abstract

The main source of microorganisms in the blood is the intestinal and oral microflora through the route of atopobiosis. It is clear that the blood microbiome undergoes significant changes in response to various pathological conditions within the human body. In this review, we summarized data from studies of the human blood microbiome in diseases of the nervous system and cardiovascular, respiratory, liver, kidney, and metabolite disorders. Links between the blood microbiome and the above mentioned diseases are demonstrated. In support of this understanding, it is evident that analogous alterations in microbiome composition occur across various disease categories; however, the microbial signatures associated with the blood microbiome exhibit specificity. For instance, an elevated abundancy of *Proteobacteria* has been identified in cardiovascular, renal, and metabolic disorders. Conversely, while *Firmicutes* are found to be abundant in renal and metabolic conditions, their levels are diminished in cardiovascular diseases. Additionally, patients suffering from respiratory and liver ailments typically show a heightened presence of *Bacteroidetes*; notably, *Flavobacterium* is prevalent in respiratory diseases, whereas *Enterobacteriaceae* is associated with liver diseases. Hence, the human blood microbiome could be considered as a potential biomarker in certain diseases.

## 1. Introduction

A well-regulated microbiome, consisting of viruses, bacteria, and fungi, is present in the bloodstream. Increasing evidence indicates that the entities of viruses, bacteria, and fungi represent living microbiota whose specific roles within the macroorganism remain largely undefined. It is believed that they gain entry into the circulatory system via the intestinal mucosal barrier [1] and the oral cavity [2]. Kalfin (1997), who reported the existence of non-pathogenic microbiota within erythrocytes [3], conducted one of the pioneering studies in this field. Following this, research has explored the microbial content in erythrocytes among both healthy individuals [4,5] and those suffering from chronic illnesses [6,7]. Recently, there has been a surge in scientific inquiry into this subject, yielding a wealth of new information. The review by Castillo et al. (2019) supports the contemporary understanding that microorganisms are normally present in the blood of healthy individuals, challenging the earlier belief that blood is sterile or that detected microbiota are merely “imported” from the intestinal and skin microbiomes [8]. An experiment by Lucchinetti et al. (2022) involving experimental mice on total parenteral nutrition further substantiates the notion that the intestinal and blood microbiomes represent distinct compartments [9]. In this context, Velmurugan et al. (2020) concluded that, in contrast to the intestinal microflora, which is predominantly composed of *Firmicutes* and *Bacteroidetes*, the blood microbiome is primarily characterized by a high abundance of *Proteobacteria* (85–90%) [10]. Sciarra et al. (2023) provide a comprehensive overview of the current understanding regarding the composition of the blood microbiome in healthy individuals, including its origins and localization within and outside the formed elements [11]. Numerous investigations have focused on the composition and variations of the human blood microbiome in response to various factors and in pathological conditions. Furthermore, a pilot study from 2024 investigated the possibility of considering the microbiome signature as a method for the identification of different body fluids (including blood) in forensic medicine [12].

Research indicates variations in the composition of the blood microbiome based on factors such as age, race, and specific conditions like smoking and pregnancy. A study conducted in 2022 examined the blood microbiome of healthy individuals, revealing age-related differences [13]. The analysis involved 37 blood samples from five families in South Korea, which demonstrated that over 95% of the blood microbiota consisted of *Proteobacteria*. Each age cohort exhibited distinct characteristics in their blood microbiome. Notably, individuals over 60 years showed a predominance of *Gammaproteobacteria*, while the classes *Alphaproteobacteria*, *Deltaproteobacteria*, and *Clostridia* were significantly less prevalent compared to younger age groups. The authors suggest that this pattern may be linked to chronic diseases commonly associated with aging, which are influenced by a microbiota that contributes to metabolic endotoxemia and chronic inflammation.

Further research by Gupta et al. (2017) identified geographical differences in the blood microbiome composition among healthy individuals based on latitude [14]. The findings indicated that populations in Germany and Poland exhibited a higher concentration of microorganisms in the central compartment, while Italy and Finland showed average levels, and Belgium and Austria had lower concentrations.

The inaugural study examining the distinct composition of the blood microbiome as a potential contributor to persistently elevated levels of interleukin-6 (IL-6) in healthy African Americans compared to Caucasian Americans was published in 2024 [15]. This investigation focused exclusively on female participants from both demographic groups, revealing the presence of eight bacterial genera/phyla. IL-6 is a critical mediator in chronic inflammatory processes, which are observed with greater frequency in the African-American population. The research team identified five bacterial genera that exhibited a significant association with IL-6 levels, notably finding that the abundance of *Actinomyces* was directly correlated with elevated plasma concentrations of this pro-inflammatory cytokine in healthy African Americans. While this research marks a promising advancement in the realm of personalized medicine, the authors did not provide epidemiological data regarding the incidence of chronic inflammatory diseases among the study participants. Additionally, a limitation of the study is its exclusive focus on female subjects.

The blood microbiome investigation conducted by You et al. (2019) spans the years 2008 to 2010 and involved 45 pregnant women who were tested two or three times throughout their pregnancy. The study identified *Firmicutes*, *Proteobacteria*, *Bacteroidetes*, and *Actinobacteria* as the predominant phyla [16]. Notably, the microbiome composition differed in women who experienced premature births, with an increase in *Firmicutes* and *Bacteroidetes*, while *Proteobacteria* exhibited a decrease. In instances of premature birth, a microbiome rich in *Bacteroides*, *Lactobacillus*, *Sphingomonas*, *Fastidiosipila*, *Weissella*, and *Butyriciococcus* was documented, leading researchers to propose that these specific taxonomic groups may be linked to the complications associated with premature delivery.

A 2021 study conducted a comparative analysis of the blood microbiome profiles of former and current smokers, marking a significant advancement in this field of research [17]. The authors assert that this comprehensive investigation will elucidate the connections between the microbiome and various pathological processes in humans, thereby enhancing the potential for personalized therapeutic approaches.

In our experience the blood microbiome of healthy individuals consists of non-harmful blood microbiota. Blood microbiota could be defined as microbial forms (L-forms, cell-wall deficient, dormant or latent microbiota) that reside primarily within the blood cells and which could be resuscitated under conditions of stress. This definition reflects widely accepted current experience. As a controversial topic, it has limitations that require further experimental clarification.

We experimentally demonstrated that the blood microbiome is viable and could be resuscitated [18,19]. Additionally, in two studies conducted by our team, we performed microscopic characterization of blood microbiota cultivated in a cell-free environment [18,20]. Demonstrating the viability and cultivability of the blood microbiota has been our strategic line of research for the past two decades. We have induced the resuscitation of latent/dormant microbial cells in blood samples from healthy individuals using temperature and chemical stress conditions. Our efforts have definitively demonstrated that bacteria and fungi are resident microbiota of blood, can be resuscitated and studied by light and electron microscopy, and can be sequenced. We quantitatively measured the culturable part of the blood microbiota of healthy individuals by culturing freshly drawn blood in a Brain Heart Infusion (BHI) medium supplemented with 10% sucrose and a high concentration of vitamin K (1 mg/mL) and incubated at 43 °C for 24 h. The explosive growth of microbial structures was observed by light microscopy on Gram-stained slides. To identify the culturable part of the blood microbiota, we applied targeted sequencing of 16S rDNA and internal transcribed spacer (ITS) markers. Dominant bacterial phyla among non-cultured samples were *Proteobacteria* 93%, *Firmicutes* 2%, *Actinobacteria* 2%, and *Planctomycetes* 2%, while among cultured samples *Proteobacteria* were 48%, *Firmicutes* 26%, *Actinobacteria* 17%, *Bacteroidetes* 4%, and *Cyanobacteria* 3%. The fungi phyla *Basidiomycota*, *Ascomycota*, and unidentified fungi were 65%, 18%, and 17%, respectively, among non-cultured samples, while among cultured samples they were 58%, 22%, and 20%, respectively. Our studies fill a knowledge gap and provide an analysis of the cultivability of circulating blood microbiota. Our group was the first to demonstrate resuscitation and measure the cultivability of blood microbiota. We first demonstrated the presence of a rich biodiversity of fungal microbiota in the blood of healthy individuals [18]. Testing new stress culture conditions, chemicals, and media should be the main strategy for optimization and better characterization of blood microbiota in healthy and diseased individuals.

The concept of a specific blood microbiome is a relatively new and contentious topic in microbiology. One of the primary arguments against the existence of a blood microbiome is the potential for contamination. Low-biomass samples like blood are highly susceptible to contamination from the skin, laboratory environments, and reagents, which can lead to false-positive results [21,22,23]. This makes it difficult to distinguish between true blood microbiota and contaminants. Some researchers propose that the microbes detected in blood are not permanent residents but transient microorganisms that enter the bloodstream from other body sites such as the gut, oral cavity, or through external factors like trauma or surgery [21,24]. This transient nature questions the concept of a stable, resident blood microbiome. There is debate over whether the microbes detected in blood are viable and capable of growth. While some studies have isolated culturable bacteria from blood, others suggest that the detected microbial DNA may represent non-viable or dormant forms of bacteria [19,21]. This inconsistency in the findings adds to the skepticism.

The study of Tan et al. (2023) has identified microbial DNA in the blood of healthy individuals, challenging the traditional view of blood as a sterile environment [25]. The research involving 9770 healthy individuals found 117 microbial species, primarily commensals from the gut, mouth, and genitourinary tract, suggesting transient and sporadic translocation rather than a consistent core microbiome.

On the contrary, another study using next-generation sequencing and metagenomics confirmed the presence of a diverse blood microbiome, dominated by *Proteobacteria*, *Firmicutes*, *Actinobacteria*, and *Bacteroidetes* [11,19]. The scientists involved explored the culturable part of the blood microbiota, identifying specific bacterial and fungal species that can be cultured under certain conditions. For instance, species from the orders *Bacillales*, *Lactobacillales*, and *Corynebacteriales* showed good cultivability, while others remained non-culturable. This diversity was also observed in different blood fractions, with the buffy coat containing the majority of bacterial DNA [26]. Several studies have confirmed the presence of a diverse microbiome in the blood of healthy individuals, primarily composed of bacteria from the *Firmicutes*, *Actinobacteria*, *Proteobacteria*, and *Bacteroidetes* phyla [11,20,26]. These findings challenge the traditional view of blood sterility [25] and suggest that blood microbiome dysbiosis could be linked to various diseases.

Although there is growing evidence that suggests the presence of a blood microbiome, considerable skepticism persists, primarily due to worries regarding contamination, the temporary nature of microbes, and various technical constraints. Certain studies have demonstrated the existence of a core blood microbiome, pinpointing specific bacterial phyla in healthy individuals [11,27,28]. These results indicate the possible existence of a blood microbiome, although it is characterized by considerable variability and a risk of contamination. The main counterargument arises from the traditional perspective on blood sterility, the risks of contamination, and the ephemeral presence of identified microbes. Moreover, the absence of reliable culturable evidence and the technical difficulties linked to next-generation sequencing (NGS) have further intensifed the discussion [21,23,29].

A novel concept of the human blood microbiome, which has traditionally been viewed as a sterile environment, has been recently explored and detailed the identification of a flourishing microbiome in healthy blood, primarily composed of four significant phyla: *Firmicutes*, *Actinobacteria*, *Proteobacteria*, and *Bacteroidetes* [11]. This microbiome is mainly found within particular populations of blood cells, especially peripheral blood mononuclear cells and erythrocytes, suggesting a complex relationship between these microorganisms and the host’s immune system. The microbiome’s predominant localization in peripheral blood mononuclear cells and erythrocytes indicates a possible origin from the skin, oral cavity, and gut. The review emphasizes the necessity of comprehending blood microbiome dysbiosis, which could act as a prognostic indicator for various diseases, especially cardiovascular disorders. Sophisticated methods such as 16S rRNA gene sequencing and mass spectrometry are being employed to investigate the blood microbiome and its metabolites. The authors utilize a theoretical framework rooted in microbiology, immunology, and systems biology. This interdisciplinary methodology facilitates a thorough understanding of the composition, function, and interactions of the blood microbiome with the host immune system. The advantages of this framework lie in its capacity to amalgamate various biological concepts and its potential to uncover new therapeutic targets. The primary assertion of this review is that the blood microbiome significantly influences health and disease. Gaining insight into its composition and dysbiosis may facilitate the development of personalized diagnostic and therapeutic strategies. Nonetheless, challenges may emerge due to the intricate nature of microbiome interactions, which can complicate the establishment of definitive conclusions regarding causality and mechanisms. This framework bolsters the authors’ argument by offering a foundation for investigating the consequences of microbiome dysbiosis in different diseases.

Sciarra et al. (2023) employ a diverse range of evidence, which encompasses recent research on the composition of the blood microbiome, microbial classification, and the impact of dysbiosis on the health of the host [11]. This comprehension is additionally reinforced by the study conducted by Tsafarova et al. (2023), which examined the morphology of the blood microbiome in healthy individuals [20].

Latent infections, such as tuberculosis, are known to be triggered by microbial species that remain uncultured during the latent phase. This latent state can persist for many years, sometimes even decades. Research has demonstrated that various viruses can exist in either a lytic or latent form within the blood and tissues throughout an individual’s lifetime [30,31]. It is reasonable to propose that for numerous microbial species, this represents a strategy to inhabit the blood or tissues of the host organism without inducing disease. Under conditions of stress, these latent infections may become active, leading to the proliferation of the persistent microbial species.

A comprehensive investigation of the blood microbiome has the potential to yield significant scientific insights that could enhance the management of human health. Currently, our understanding of dysbiosis within this central compartment is markedly limited. Such dysbiotic conditions are frequently linked to a suspected infectious origin that remains unexplained. Notably, diseases like schizophrenia, amyotrophic lateral sclerosis, and cancer may be correlated with the pathological state of the blood microbiome [32].

**Figure 1:**
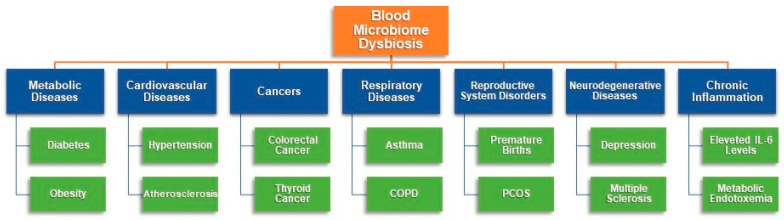
Examples of pathological conditions linked to blood microbiome dysbiosis.

Numerous studies have emerged over the past decade that investigate various dimensions of this hypothesis. These studies highlight certain inconsistencies and address the limitations of the proposed concepts. It is clear that the blood microbiome undergoes significant changes in response to various pathological conditions within the human body as illustrated in Figure 1.

These alterations have been observed across multiple systems, including the gastrointestinal [33,34,35,36], cardiovascular [37,38,39], respiratory [17], excretory [40,41,42], and reproductive systems [23,43]. Additionally, changes in the blood microbiome have been linked to type 2 diabetes [44] and various malignancies [45,46].

There is a compilation of data regarding the composition of the circulating microbiome, particularly concerning the presence of dysbiosis in patients with non-infectious conditions such as diabetes and cardiovascular diseases, aimed at enhancing the understanding of early detection and the pathogenic mechanisms involved [21]. Additionally, another review article explores the association between dysbiotic blood microbiota (specifically L-type or cell wall-deficient microbes) and various diseases, including multiple sclerosis, Parkinson’s disease, psoriasis, diabetes, and thyroid cancer, considering its potential to trigger disease and serve as a prognostic model [47].

Pilot studies have been undertaken to explore the potential mechanisms underlying the interactions between microbiota and macroorganisms. Microorganisms engage with the cells of a healthy host through various compounds produced during circulation, including metabolites, lipoglycans, peptides, proteins, and bacterial extracellular vesicles [48]. In their comprehensive review, Jensen and Benson (2019) discuss the hypothesis that the composition of the circulating microbiome differs in patients with chronic diseases compared to that of healthy individuals [49]. Supporting this notion, Luo et al. (2021) conducted a study aimed at identifying an appropriate method for detecting alterations in the plasma microbiome of immunocompromised patients, thereby enhancing the qualitative application of this approach in clinical settings [50]. Recent advancements have enabled the monitoring of changes in the blood microbiome composition, which may serve as diagnostic and prognostic indicators in clinical practice [51]. Accumulated clinical evidence from various conditions, including early-stage cardiovascular diseases [38,52], type 2 diabetes [37], and non-alcoholic fatty liver disease in obese individuals [33], suggests that alterations in the blood microbiome could function as biomarkers.

A significant fraction of blood metabolites (64%) is linked to either host genetics or the gut microbiome. Importantly, 69% of these connections are exclusively attributed to the microbiome, underscoring the considerable impact of microbial activity on the blood metabolome [53]. Metabolites generated by the gut microbiota, including short-chain fatty acids (SCFAs), indoles, and lipopolysaccharides, can permeate the bloodstream and influence overall health. The effects of these metabolites can be both advantageous and harmful, contingent upon their concentration and the specific circumstances surrounding their presence [54,55]. Certain microbial metabolites, such as trimethylamine N-oxide and lipopolysaccharides, are associated with endothelial dysfunction, the development of atherosclerosis, and elevated blood pressure. In contrast, short-chain fatty acids (SCFAs), including acetate, propionate, and butyrate, serve protective functions by promoting homeostasis and mitigating inflammation [53,55].

The impact of the gut microbiome on the blood metabolome is also apparent in various metabolic disorders. For example, particular microbial metabolites have been linked to obesity, type 2 diabetes, and non-alcoholic fatty liver disease, indicating that microbial metabolic pathways are integral to the pathophysiology of these conditions [56]. Moreover, dietary choices have a profound impact on the gut microbiome, which in turn influences the blood metabolome. For instance, fiber-rich diets promote the proliferation of beneficial microorganisms that generate SCFAs, which are then absorbed into the bloodstream and play a vital role in metabolic health [57,58]. The administration of antibiotics modifies the gut microbiome, resulting in alterations to the blood metabolome. Certain antibiotics can specifically influence the concentrations of important metabolites such as hippuric acid and indole-3-acetic acid, thereby underscoring the microbiome’s significance in metabolite synthesis [59].

The blood microbiome significantly impacts the metabolome, influencing various health conditions and disease processes. This relationship highlights the necessity of integrating microbiome considerations into both diagnostic and therapeutic frameworks to leverage its potential for enhancing human health. The connection between the microbiome and the blood metabolome presents diagnostic opportunities; for example, distinct metabolite profiles in the blood may reflect changes in gut microbiota, which can aid in the diagnosis of diseases such as colorectal cancer [60,61]. A deeper understanding of microbiome–metabolome interactions paves the way for targeted therapeutic strategies. By modifying the gut microbiome through dietary changes, probiotics, or other interventions, it may be possible to influence the blood metabolome and improve health outcomes [62,63].

Imbalances in the blood microbiome have a profound effect on the metabolome, affecting numerous metabolic pathways and playing a role in the development and advancement of several diseases. This connection is observable across a range of health issues and through various mechanisms.

Dysbiosis has a significant impact on the synthesis and uptake of metabolites. For example, the use of antibiotics that modify the composition of gut microbiota results in alterations in plasma metabolites, including hippuric acid, indole-3-acetic acid, and glycerol, along with various amino acids and carbohydrates [57,59]. This suggests that the microbiome’s ability to produce metabolites that are absorbed by the host is impaired in the state of dysbiosis.

Dysbiosis may contribute to systemic inflammation and metabolic disorders. In Table 1 the altered microbiota impact has been summarized. In the context of chronic liver diseases, dysbiosis is associated with heightened gut permeability and systemic inflammation, subsequently influencing the synthesis of SCFAs and bile acids, thereby modifying the host’s metabolic processes [64,65]. Likewise, in cases of hypertension, dysbiosis is linked to changes in plasma metabolites, including sphingosine 1-phosphate, which plays a role in the regulation of blood pressure [66].

Distinct diseases exhibit specific alterations in metabolomic profiles as a result of dysbiosis. In the case of chronic hepatitis B, dysbiosis induces modifications in gut microbiota and metabolite levels, which in turn influence peripheral immune responses and the advancement of the disease [67]. Similarly, in myositis, dysbiosis correlates with variations in inflammatory markers and serum metabolites, including glutamate and taurine [68].

Dysbiosis affects the gut–brain axis, thereby influencing mental health via metabolic pathways. Prolonged use of antibiotics, which causes dysbiosis, modifies the gut microbiota and metabolite profiles, resulting in alterations in brain-signaling molecules and behaviors associated with anxiety [69].

Dysbiosis elicits distinct responses based on sex. For example, in patients suffering from trauma and sepsis, dysbiosis results in sex-specific alterations in fecal metabolites, while changes in plasma metabolites remain consistent across both sexes [70].

**Figure 2:**
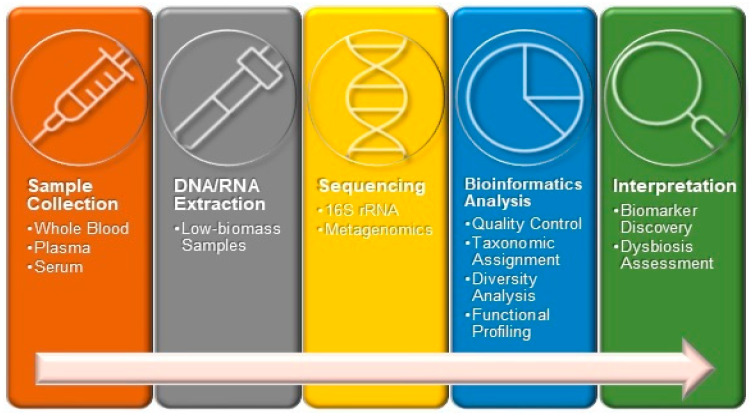
The process of blood microbiome analysis.

An increasing body of research is focused on the alterations in the blood microbiome, specifically dysbiosis, associated with particular diseases or disease categories. A variety of reviews has been released that explores different aspects of this topic, including the application of dysbiosis in the blood microbiome as a potential biomarker for certain diseases [11]. Additionally, these reviews address the difficulties associated with microbiota detection and the need for standardized methodologies [23]. Furthermore, they provide insights into the characterization of both eubiotic and dysbiotic conditions of the microbiome found in the bloodstream [71]. The current approach to conducting blood microbiome composition is presented in Figure 2.

Enhancing the expertise of professionals in this area could prove beneficial not only for the prediction and monitoring of pathological conditions but also for evaluating the risks associated with blood transfusions. This review aims to consolidate recent findings to enhance the comprehension of pathological mechanisms in relation to circulating microbiotas. The necessity for a comprehensive summary of current knowledge is evident, particularly following a mini-review published in 2020 [72] and an editorial from 2023 [73], both of which referenced only a limited number of sources.

## 2. Results

Microbiota may infiltrate the bloodstream via the oral and intestinal mucosal barriers. This process of translocation is frequently linked to heightened intestinal permeability, which can be affected by various factors including dietary habits, lifestyle choices, and pathological conditions [74,75]. Changes in the gut microbiome may weaken the intestinal barrier, permitting detrimental substances and pathogens to infiltrate the circulatory system [75,76,77]. When introduced into the bloodstream, microbiota and their byproducts, such as lipopolysaccharides, can initiate an immune response. This response encompasses the synthesis of cytokines and inflammatory mediators, integral components of the body’s defense system [78,79,80]. The presence of endotoxins in the circulatory system can trigger systemic inflammation and activate the immune response, potentially leading to the eradication of certain microorganisms [79,80].

Notwithstanding the immune response, research indicates that microorganisms are capable of persisting in the bloodstream. Investigations have revealed a variety of microbial communities present in the blood of different patient groups, including individuals who exhibit no clinical symptoms of infection [74]. The existence of circulating microbiota has been associated with chronic inflammatory diseases, indicating that certain microorganisms may remain in the bloodstream and play a role in the development of these conditions [74,75]. The persistence of microbiota within the bloodstream is shaped by the intricate relationships between the host’s immune system and microbial metabolites. Such interactions have the capacity to alter immune responses, thereby enabling certain microorganisms to avoid prompt eradication [78,81,82]. Metabolites generated by the microbiota can influence immune responses and may contribute to the sustained presence of microorganisms in the bloodstream [82,83].

Microbiota may infiltrate the bloodstream due to weakened gut barriers and heightened intestinal permeability. Once in circulation, the immune system may respond by attempting to eradicate these microorganisms through inflammatory mechanisms. Nevertheless, certain microorganisms are capable of surviving and remaining in the bloodstream, which can lead to chronic inflammation and the development of diseases. The persistence and effects of circulating microbiota are shaped by the complex interactions between microbial byproducts and the host’s immune response [74,75,78,79].

### 2.1. Changes in Blood Microbiome in Nervous System Disorders

The central nervous system is of extreme importance for the appropriate functioning of the whole body. The brain is well-protected by the blood–brain barrier which excludes most microbes. Nevertheless, bacteria can pass through the gut barrier and can be found in the blood. In this regard, recently it was demonstrated that extracellular vesicles, produced by gut microbiota, can enter systemic circulation and be delivered to body sites, including the brain [84]. Hence, it is not surprising that the blood microbiome was shown to be involved in some nervous system disorders. It is interesting to note that an oral bacterium, *Porphyromonas gingivalis*, was detected as translocating to the brain and playing a role in Alzheimer’s disease [85].

Serum microbiome composition may be an useful tool in distinguishing between major depressive disorder and bipolar disorder [86]. In particular, Rhee and coworkers (2020) demonstrated that *Ruminococcaceae* UCG-002 and *Prevotella* 2 genera might be used for distinguishing the above-mentioned depressive disorders. Inspired by the results, the team continued research on this topic, and found out that anxiety symptoms were associated positively with *Desulfovibrionaceae* family DNA composition in the serum of patients [87]. On the other hand, the *Desulfovibrionaceae* family was shown to be composed of gram-negative anaerobes which produce hydrogen sulfide [88]. In such a regard, the pro-inflammatory property of hydrogen sulfide could mediate the association between inflammation and anxiety severity [89]. Patients with depression were also shown to possess a distinct blood microbiome that changes after antidepressant treatment [90]. Using shallow-shotgun metagenomic sequencing to characterize the plasma microbiome of people with human immunodeficiency viruses and healthy controls, all of whom underwent a comprehensive neuropsychiatric assessment, Taylor and coworkers demonstrated that the risk of major depressive disorder might be increased by circulating plasma microbiome in people with human immunodeficiency viruses [91]. Recently, transcriptome analysis in whole blood was shown to reveal increased microbial diversity in schizophrenia [92]. In this interesting study, by using high-quality RNA sequencing reads, the authors investigated the blood microbiome in patients with bipolar disorder, amyotrophic lateral sclerosis, and schizophrenia, as well as in healthy controls. As a result a wide range of bacterial and archaeal phyla were detected in the blood. Increased microbial diversity was demonstrated in patients with schizophrenia as compared to those with amyotrophic lateral sclerosis or bipolar disorder or healthy individuals [92].

Obsessive–compulsive disorder (OCD) is often considered as a highly disabling neuropsychiatric disorder which can impair the general functions of an individual. Kang and coworkers (2023) examined bacterial DNA in serum samples taken from OCD patients and healthy controls [93]. They found that *Pseudomonas*, *Caulobacteraceae (f)*, *Streptococcus*, *Novosphingobium*, and *Enhydrobacter* at the genus level were significantly less prevalent in patients with OCD than in healthy controls. In addition, among patients with OCD, the microbial composition in the early-onset versus late-onset types was significantly different with respect to the genera *Corynebacterium* and *Pelomonas*.

Links between multiple sclerosis and the human blood microbiome were studied by examining the change in the representation of microbiota phylotypes, the proportion of coccal flora, the proportion of anaerobic, gram-negative, proteolytically active microflora, as well as the concentration of markers of bacterial plasmalogen and endotoxin in the blood [94]. It was shown that the proportion of coccal, gram-negative, anaerobic microflora with a proteolytic type of metabolic activity increases in multiple sclerosis. This elevation in blood concentrations of microbial markers of bacterial plasmalogen and endotoxin may be associated with an increase in the permeability of the intestinal wall. Hence, multiple sclerosis could be associated with pathological changes in the structure of the microbiome, which might be among important factors in the pathogenesis of this disease.

### 2.2. Changes in Blood Microbiome in Cardiovascular Diseases

Cardiovascular diseases (CVD) represent a significant public health concern, impacting a substantial segment of the global population. This category encompasses conditions such as hypertension, hyperlipidemia, atherosclerosis, and heart failure, along with associated complications. These diseases not only pose risks of severe health complications and mortality but are also frequently linked with co-morbidities involving metabolic, renal, and other disorders. The expression and prognosis of these conditions are influenced by a variety of factors. Recently, there has been growing interest within the scientific community regarding the microbiome’s influence on the pathological mechanisms that affect cardiovascular health. A study conducted by Guo et al. (2021), which synthesizes findings from 17 studies with over 9000 participants, explores the connection between hypertension and the intestinal microbiome [95]. The proliferation of certain *Proteobacteria* and *Bacteroidetes* has been associated with the enhanced production of lipopolysaccharides, the activation of specific transporters, and the inhibition of the metabolism of various amino acids. This phenomenon is indicative of dysbiosis within the intestinal microbiota, characterized by diminished diversity, alterations in microbial composition and functionality, changes in microbial interactions, and other contributing factors.

Over a decade ago, Amar et al. (2013) explored the potential implications of the blood microbiome in the context of cardiovascular diseases [37]. Their research, which encompassed nearly 4000 patients over a nine-year follow-up period, identified dysbiosis of the blood microbiota as an independent risk factor for the onset of heart disease. For those who experienced cardiovascular complications during the study, a notable reduction in the normally present microbiota, alongside an increase in *Proteobacteria* levels, was observed. Rajendhran et al. (2013) elucidated the distinct composition of the blood microbiome in healthy individuals versus those suffering from cardiovascular diseases in India [96]. In healthy subjects, the predominant bacterial phyla are *Firmicutes* and *Proteobacteria*. Conversely, patients exhibited a notable dysbiosis, characterized by a significant increase in *Proteobacteria*, particularly from the family *Pseudomonadaceae* and class *Gammaproteobacteria*, alongside a reduction in *Firmicutes*, specifically from the family *Staphylococcaceae*. Supporting this observation, Koren et al. (2011) reported a similar microbiota composition within atherosclerotic plaques [97]. Additionally, Sharifullina et al. (2023) investigated the circulating microbiome in individuals with atherosclerotic vascular damage, involving a cohort of 35 patients (23 men and 12 women) diagnosed with carotid artery atherosclerosis [98]. The bacterial cultures derived from both blood samples and atherosclerotic plaques exhibited slow growth on nutrient media, with the growth rate correlating with leukocyte counts and high-density lipoprotein levels in the participants’ blood. According to the findings of Dinakaran’s team [38], patients with CVD exhibited elevated levels of circulating bacterial DNA and greater bacterial diversity compared to healthy individuals. Specifically, in the cohort of CVD patients (*n* = 80), *Actinobacteria* were the most prevalent phylum, followed by *Proteobacteria* [38]. In contrast, healthy controls (*n* = 40) showed a predominance of *Proteobacteria*, with *Actinobacteria* following. Furthermore, while healthy individuals demonstrated a higher prevalence of eukaryotic viruses, particularly *Lymphocystis virus* and *Torque Teno viruses*, the virome in CVD patients was notably enriched with bacteriophages, primarily those associated with *Propionibacterium*, *Pseudomonas*, and *Rhizobium*.

In their studies conducted in 2020 and 2022, Velmurugan et al. highlighted the significant role of the human microbiome in the development and progression of various cardiovascular and metabolic disorders [10,99]. They substantiated the hypothesis that dysbiosis and a reduction in the diversity of blood microbiota are prevalent in conditions that impact the cardiovascular system and metabolic health. The authors suggest that these findings may facilitate the integration of microbiome-focused strategies into therapeutic interventions. Additionally, Jing et al. (2021) examined the correlation between the composition of the peripheral microbiome and the likelihood of developing hypertension [100]. Their research involved 150 individuals diagnosed with hypertension and 150 healthy participants, carefully matched for sex and age. The results indicated a higher prevalence of *Proteobacteria* and a lower prevalence of *Firmicutes* and *Bacteroidetes* in the hypertensive group compared to the control group without hypertension.

Khan’s research team published a series of studies in 2022 that provided an in-depth analysis of the alterations in the blood microbiome associated with various nosological conditions impacting the cardiovascular system. One notable study focused on the influence of circulating microbiota on atherosclerotic damage [101]. This investigation involved 70 patients diagnosed with acute coronary syndrome, 70 with chronic coronary syndrome, and 70 healthy control subjects from China. The findings revealed an increase in microbiota diversity among patients with acute coronary syndrome, whereas a decrease was observed in those with chronic conditions. The blood microbiome exhibited distinct compositions across the three participant groups, with the microbial profiles of both acute and chronic coronary syndrome patients differing significantly from that of the healthy cohort. This supports the hypothesis that the microbial ecology within the blood undergoes alterations in pathological states. At the phylum level, the blood microbiota of patients with acute coronary syndrome, chronic coronary syndrome, and healthy individuals was predominantly composed of *Firmicutes* (39%, 45%, 43%, respectively), *Bacteroidetes* (31%, 32%, 29%, respectively), and *Proteobacteria* (19%, 15%, 15%, respectively). Comparative analysis between the heart disease groups indicated that *Proteobacteria* were overrepresented in acute coronary syndrome patients (19% vs. 9%), while *Firmicutes* (44% vs. 38%) and *Bacteroidota* (32% vs. 31%) were found to be underrepresented in this group relative to those with chronic disease. In the blood of patients with acute coronary syndrome, a notable increase in the phylum Acidobacteriota was observed, followed by *Actinobacteriota*, *Cloacimonadota*, *Fibrobacterota*, *Fusobacteriota*, *Latescibacterota*, *Myxococcota*, *Nitrospirota*, *Proteobacteria*, *Synergistota*, and *Thermotogota*. Conversely, there was a marked reduction in the levels of *Cyanobacteria*, *Deferribacterota*, *Desulfobacterota*, *Firmicutes*, *Gemmatimonadota*, *Patescibacteria*, and *Verrucomicrobiota* when compared to individuals with chronic coronary syndrome. A comparative analysis of the blood microbiome composition between affected patients and healthy controls further substantiates the hypothesis that such evaluations may yield potential biomarkers for the identification of coronary diseases. In the cohort with acute coronary syndrome, *Bacteroidota* and *Proteobacteria* emerged as the most prevalent phyla, whereas Firmicutes predominated in the healthy control group. In contrast, the chronic coronary syndrome group exhibited a slight increase in *Firmicutes* and *Bacteroidota*, alongside a decrease in *Proteobacteria* relative to healthy volunteers. The researchers later broadened their investigation to include patients diagnosed with myocardial infarction (*n* = 29) [102]. Their findings revealed a notable reduction in microbial diversity within the myocardial infarction cohort when compared to healthy controls (*n* = 29). Additionally, there were significant differences in both the composition and richness of the microbiota between the infarction patients and the healthy subjects. Notably, members of the *Actinobacteria* phylum (class *Actinobacteria*, order *Bifidobacteriales*, family *Bifidobacteriaceae*, genus *Bifidobacterium*) were found to be significantly more prevalent among the myocardial infarction patients, whereas the *Bacteroidetes* phylum (class *Bacteroidia*, order *Bacteroidales*) was more dominant in the healthy control group. A subsequent study conducted in 2024 further substantiated the notion that alterations in the blood microbiome could serve as an independent biomarker for acute myocardial infarction [103]. This investigation involved 55 patients with acute myocardial infarction and 62 patients with unstable angina. The results indicated a significantly higher diversity of the serum microbiome in the infarction group compared to those with unstable angina. Conversely, the unstable angina patients exhibited a greater abundance of *Gammaproteobacteria*, *Proteobacteria*, *Ralstonia pickettli*, *Ralstonia*, *Burkholderiaceae*, and *Burkholderiales*, while the infarction group showed higher levels of *Bacteroidales*, *Bacteroidia*, *Bacteroidota*, *Clostridia*, and *Firmicutes*.

In addition, other diseases within this category impact the vascular wall. In 2021, Desbois et al. identified a distinct microbiome signature in patients suffering from inflammatory diseases affecting large vessels, specifically Takayasu arteritis and giant cell arteritis [104]. This investigation included 13 patients with Takayasu arteritis, 9 with giant cell arteritis, and 15 healthy controls. The findings revealed elevated levels of *Clostridia*, *Cytophagia*, and *Deltaproteobacteria*, along with a reduction in *Bacilli* among patients with Takayasu arteritis. Conversely, those diagnosed with giant cell arteritis exhibited an increased presence of *Rhodococcus* and an unidentified member of the *Cytophagaceae* family. Notably, the microbiota in Takayasu arteritis patients displayed higher levels of *Candidatus Aquiluna* and *Cloacibacterium* compared to those with giant cell arteritis.

Lawrence et al. (2022) established a connection between the composition of the circulating microbiome and the risk of mortality due to cardiovascular disease [39]. Their case-cohort study spanned nine years and involved age-matched participants (*n* = 227) who succumbed to cardiovascular disease, alongside controls who were randomly selected from the same cohort (*n* = 178). A limitation of this research is that it exclusively included male participants. The study identified three bacterial genera that were significantly correlated with mortality as independent factors: elevated levels of *Kocuria* and *Enhydrobacter* were associated with increased cardiovascular mortality, whereas Paracoccus exhibited an inverse relationship.

Notable alterations in microbial communities within the blood, gastrointestinal tract, and oral regions have been identified in patients with myocardial infarction (MI), revealing unique microbial profiles in each compartment [24]. This research examines the microbial diversity in 144 patients with myocardial infarction (MI) in contrast to 24 healthy controls, employing 16S rRNA sequencing techniques. Notable alterations in microbial populations were identified, with MI patients exhibiting an increase in phyla such as *Armatimonadota* and *Caldatribacteriota* in their blood, as well as genera including *Bacillus*, *Pedobacter*, and *Odoribacter*. These findings suggest a unique microbial profile in the bloodstream associated with MI.

Table 2 provides a schematic representation of the characteristics of the microbiome in individuals diagnosed with CVD.

### 2.3. Changes in Blood Microbiome in Respiratory Diseases

Research concerning the influence of the microbiome on lung disease has predominantly concentrated on the specific characteristics of the gut microbiome [105,106,107,108]. The investigation of the local microbiome within the respiratory tract, particularly in relation to pathological processes that impact the respiratory system, represents a relatively novel area of research [109,110,111,112].

At present, there is a scarcity of literature examining the changes in the microbiome plasma signature linked to diseases of the respiratory system. The beforementioned study conducted by Morrow et al. (2021) involved a sample of 2590 participants, which included both individuals who had previously smoked and those who were current smokers, as well as individuals with and without chronic obstructive pulmonary disease (COPD) [17]. The predominant bacterial phyla identified in the blood samples included *Proteobacteria*, *Actinobacteria*, *Firmicutes*, and *Bacteroides*. Notable correlations were observed between the severity of dyspnea and the presence of *Acinetobacter*, *Serratia*, *Streptococcus*, and *Bacillus* in the bloodstream. In active smokers, the blood microbiome was primarily characterized by the dominance of *Acinetobacter*, *Serratia*, and *Cutibacterium*. In the preceding year, Morrow et al. released findings concerning alterations in the blood microbiome that correlate with the clinical manifestations of chronic obstructive pulmonary disease [113]. The occurrence of *Flavobacterium* has been linked to both the onset and advancement of emphysema. Additionally, a correlation was identified between the exacerbation of COPD and the relative prevalence of *Staphylococcus*, *Acidovorax*, and *Cupriavidus*.

A research investigation conducted in the same year revealed variations in the blood microbiome and suggested these differences as a possible diagnostic indicator for asthma [114]. A total of 260 healthy participants and 190 individuals diagnosed with asthma were recruited for this study. Notable differences were observed in the blood microbiome between asthma patients and healthy controls. Specifically, at the phylum level, *Bacteroidetes* exhibited a higher prevalence in individuals with asthma, whereas *Actinobacter*, *Verrucomicrobia*, and *Cyanobacteria* were found to be more abundant in the healthy control group.

A separate investigation examined the microbiome signature present in the blood, gastrointestinal tract, and lungs of individuals suffering from acute respiratory distress syndrome [115]. Blood samples from a cohort of 16 patients, collected over the course of one year, were analyzed. The study revealed a 20% overlap in the microbiota composition across the three examined compartments. Notably, the presence of the *Proteobacteria* phylum was observed in the blood of healthy individuals, correlating with reduced inflammation levels in the clinical cases assessed. Conversely, *Bacteroidetes* emerged as the predominant phylum within the blood microbiome of patients suffering from acute respiratory distress syndrome. It is important to note that the pilot study is constrained by a limited sample size, as well as other methodological considerations, rendering the findings more suggestive than statistically robust.

### 2.4. Changes in Blood Microbiome in Liver Damage

The liver plays a crucial role in the survival of the organism. Its strategic position and diverse functions render it susceptible to various diseases, which can arise from different causes, including infectious, toxicological, and parasitological factors. These conditions are frequently associated with immune responses, inflammatory reactions, or metabolic alterations.

The hypothesis linking microbiome composition to overall health has been recognized in scientific discourse for some time. However, it was not until 2016 that two separate studies were published examining the relationship between the blood microbiome and individuals suffering from liver cirrhosis and obesity by Lelouvier et al. (2016) [33], as well as those with liver cirrhosis and ascites by Santiago et al. [116]. The research conducted by Lelouvier et al. which involved 50 patients from Spain and 71 from Italy, identified an increased presence of microorganisms in patients with liver fibrosis, yet it did not succeed in pinpointing a distinct microbiome signature. Santiago et al. [116] conducted a study examining the microbiome composition of intestinal, blood, ascitic, and fecal microflora in patients with liver cirrhosis accompanied by ascites (*n* = 13), in comparison to those with cirrhosis without ascites (*n* = 14). Their findings revealed that the serum microbiome of individuals with ascites exhibited elevated levels of lipopolysaccharide-binding protein, a known indicator of microbial translocation. This phenomenon accounts for the increased diversity and relative abundance of *Clostridiales* and an unidentified genus within the phylum *Cyanobacteria*, alongside a diminished presence of *Moraxellaceae* in patients with ascites relative to those without. The authors suggest that alterations in the blood and fecal microbiome composition may serve as biomarkers for assessing the progression of cirrhosis. Building on the work of Santiago et al. [116], a subsequent study conducted in the following year by Traykova et al. (2017) focused on the blood microbiome of patients with decompensated cirrhosis (*n* = 9) compared to healthy controls (*n* = 9) [34]. This investigation revealed a significant increase in the number of bacterial species present in the blood of cirrhotic patients compared to the control group. Specifically, 9 out of 12 bacterial orders were identified in individuals with liver disease, whereas only 2 were detected in healthy subjects. The microbiota in cirrhotic patients included the following types: *Firmicutes* (nine species), *Proteobacteria* (six species), *Bacteroidetes* (three species), and *Verrucomicrobia* (one species). However, the study’s limitations include the small sample size and the exclusive inclusion of male participants. Conversely, the research was enhanced by exploring the correlations between the identified microbiota and various hemodynamic parameters and inflammatory markers. A study conducted in 2019 analyzed the blood microbiome composition of 66 patients suffering from liver cirrhosis alongside 14 healthy individuals [35]. The investigation revealed that patients exhibited 183 distinct taxonomic units at the genus level, in contrast to the 123 units identified in the healthy cohort. Notably, the blood microbiome of individuals with the liver disease showed a marked increase in *Enterobacteriaceae*, whereas the levels of *Akkermansia* (phylum *Verrucomicrobiota*), *Rikenellaceae* (Phylum: *Bacteroidota*), and *Erysipelotrichales* (phylum *Bacillota*) were significantly diminished when compared to the healthy controls.

A study conducted in 2024 established a correlation between the progression from compensated to decompensated liver cirrhosis in hepatitis C patients and alterations in blood microbiome composition [117]. This investigation included a total of 88 subjects. The findings revealed that individuals with decompensated cirrhosis exhibited a significant reduction in microbial diversity, characterized by an increased prevalence of *Proteobacteria*, *Alphaproteobacteria*, *Sphingomonadales*, and *Sphingomonadaceae* when compared to those with compensated cirrhosis.

Further research in this domain has been conducted, including studies from 2018 that explored the blood microbiome in various liver diseases, specifically in patients suffering from severe acute pancreatitis and alcoholic hepatitis. One particular study involved 50 patients diagnosed with severe acute pancreatitis—comprising groups with no infection (*n* = 17), with infection (*n* = 16), and septic cases (*n* = 17)—alongside 12 healthy controls [118]. The objective was to monitor changes in microbial composition and to establish correlations with disease severity and the likelihood of complications. The microbiomes associated with blood and neutrophils in the pancreatitis cases were found to be enriched with *Bacteroidetes* and *Firmicutes*, while a decline in *Actinobacteria* was noted. These microbial alterations were associated with elevated serum cytokine levels and various immunological markers. The authors concluded that the observed changes in the blood microbiome during acute pancreatitis do not serve as reliable predictors for the risk of infectious complications. A study conducted by Puri et al. in 2018 examined patients with moderate (*n* = 18) and severe (*n* = 19) alcoholic hepatitis, comparing their results with those of alcoholics without liver disease (*n* = 19) and non-alcoholic controls (*n* = 20) [119]. The findings revealed a significant reduction in the presence of *Bacteroidetes* among all individuals who consumed alcohol when compared to the non-alcoholic controls. Conversely, all alcohol-consuming groups exhibited an increased presence of *Fusobacteria*, with the highest levels found in alcoholics without liver damage, which subsequently decreased in the groups with alcoholic hepatitis. Notably, the analysis indicated that both alcoholics without hepatitis and those with severe alcoholic hepatitis harbored gram-negative bacterial flora, suggesting that excessive alcohol consumption is a contributing factor to the alterations observed in the circulating microbiome.

Additionally, a study by Gedgaudas et al. (2022) focused on the blood microbiome variations in patients suffering from portal hypertension, analyzing its composition across two distinct vascular compartments: the peripheral and hepatic veins [120]. This investigation included 58 cirrhotic patients and 46 healthy controls, while also monitoring pH levels, liver function, inflammatory markers, and gut permeability. The findings revealed that the blood microbiome of individuals with liver damage exhibited elevated levels of *Comamonas* (Class: *Betaproteobacteria*), *Cnuella* (phylum *Bacteroidota*), *Dialister* (phylum *Bacillota*), *Escherichia/Shigella* (class *Gammaproteobacteria*), and *Prevotella* (phylum *Bacteroidota*), alongside reduced levels of *Bradyrhizobium* (class *Alphaproteobacteria*), *Curvibacter* (class *Betaproteobacteria*), *Diaphorobacter* (class *Betaproteobacteria*), *Pseudarcicella*, and *Pseudomonas* (class *Gammaproteobacteria*). The presence of genera such as *Bacteroides*, *Escherichia/Shigella*, and *Prevotella* was linked to severe clinical manifestations, with *Escherichia/Shigella* and *Prevotella* correlating with heightened interleukin-8 levels. Nevertheless, the authors concluded that the profiles of circulating microbiota do not serve as reliable predictors of disease severity.

In 2023, Vasudevan et al. examined alterations in the blood microbiome associated with various liver diseases, such as liver cirrhosis, fibrosis, hepatocellular carcinoma, chronic viral liver failure, and alcoholic hepatitis, among others [121]. The authors explored the potential of these microbiome changes as independent biomarkers for liver-related pathologies. Furthermore, rats with experimental liver cirrhosis exhibit elevated instances of bacterial translocation, defined as the migration of bacteria from the gastrointestinal tract into the circulatory system, which exacerbates systemic inflammation and leads to hepatic injury [122].

A 2024 research study examines the variations in blood microbiome and metabolome characteristics between 88 patients infected with HCV who have Child-Turcotte–Pugh class B (CTP-B) and class A (CTP-A) cirrhosis [117]. The participants were sourced from four tertiary referral hospitals in Madrid, Spain, during the timeframe of January 2015 to June 2016. The findings indicate that patients classified as CTP-B demonstrate reduced richness and diversity in their blood microbiome, alongside a notable rise in the presence of *Proteobacteria*, *Alphaproteobacteria*, and *Sphingomonadales* when compared to those in the CTP-A category.

An intriguing area of research involves the examination of microbiome dynamics in patients undergoing liver transplantation. A study conducted in 2021 monitored 51 liver transplant recipients for a duration of up to 8 weeks post-surgery [123]. The analysis revealed significant fluctuations in the blood microbiome composition, particularly concerning the levels of *Anelloviridae*, *Nocardiaceae*, *Microbacteriaceae*, and *Enterobacteriaceae* at various postoperative intervals. Notably, in patients experiencing acute cellular graft rejection, there was a marked increase in *Enterobacteriaceae* levels and a reduction in microbiome diversity, in contrast to those who did not face rejection. Additionally, a recent study from 2024 explored the potential correlation between alterations in the plasma microbiome and the onset of liver failure following partial hepatectomy [124]. Samples were obtained from 158 participants at three critical time points: preoperatively, during surgery from the portal vein prior to occlusion, and one day post-intervention. The findings indicated that patients who subsequently developed liver failure exhibited a higher bacterial load in the central compartment, along with a more diverse mycobiome prior to the surgical procedure.

The observed changes in the blood microbiome in various liver injuries are summarized in Table 3.

### 2.5. Changes in Blood Microbiome in Kidney Diseases

A significant body of the literature investigates the connection between renal disease and the human microbiome, with the majority of these studies emphasizing the importance of the gut microbiome [125,126,127,128,129,130,131,132,133,134,135]. In contrast to current understanding, Bossola and associates published findings in 2009 indicating that healthy volunteers exhibited no detectable blood microbiota. Conversely, their investigation involving 58 hemodialysis patients revealed the presence of several bacterial species, including *E. coli*, *S. aureus*, *P. aeruginosa*, *S. epidermidis*, *E. faecalis*, *P. mirabilis*, and *S. haemolyticus* [136].

Liu et al. (2020) conducted a review highlighting the significance of urinary microbiome composition within the pelvic region of individuals suffering from nephrolithiasis [137]. Furthermore, another investigation indicated that similar alterations occur in cases of acute kidney injury [138]. Additionally, various studies have tracked modifications in the urogenital [139] and oral [140,141] microbiomes associated with chronic kidney disease (CKD).

The existing knowledge regarding the connection between the blood microbiome and kidney diseases remains limited. Simoes-Silva et al. conducted a pioneering investigation in this area in 2018 [142]. The research team investigated the effects of hemodialysis and peritoneal dialysis on various microbiomes, including those of the skin, oral cavity, intestines, and peritoneum, as well as the blood microbiome, in patients suffering from chronic kidney disease. Subsequently, a pilot study detailing alterations in the blood microbiome among these patients was published the following year [40]. The research involved 20 individuals diagnosed with moderate non-diabetic chronic renal failure and 20 healthy participants. The limited sample size and the variability within the renal impairment cohort were identified as limitations of the study. Nevertheless, the findings revealed a notable reduction in α diversity, indicating a decrease in the richness of bacterial taxa within the chronic kidney disease group when compared to the healthy controls. Furthermore, there was a significant increase in the proportion of *Proteobacteria*, specifically the class *Gammaproteobacteria*, in the renal impairment group relative to the control group. Additionally, patients with CKD exhibited greater diversity within the *Enterobacteriaceae* and *Pseudomonadaceae* families, which was found to correlate with a diminished glomerular filtration rate.

The analysis conducted by Wehedy et al. (2022) explores the significance of dysbiosis within the intestinal, urinary, and blood microbiomes concerning the initiation and advancement of chronic kidney disease [143]. According to the authors, endotoxins produced by intestinal microorganisms, entering the circulation, have the potential to alter the blood microbiome, causing dysbiosis in the central compartment.

A pilot study conducted in 2022 established a connection between alterations in the gut and blood microbiomes of patients with chronic kidney failure who were receiving peritoneal dialysis. The study comprised 44 participants from Portugal, with 11 individuals exhibiting no vascular complications, while the remaining patients had experienced vascular calcification [42]. Variations in the blood microbiome composition have been observed among patients with CKD, particularly distinguishing those with vascular complications from those without. Notably, the taxa *Cutibacterium* (Class *Actinomycetia*), *Pajaroellobacter*, *Devosia* (Class *Alphaproteobacteria*), and *Hyphomicrobium* (Class *Alphaproteobacteria*) are prevalent in individuals experiencing vascular calcification. In contrast, *Pelomonas* is more frequently found in patients who do not exhibit this complication. Furthermore, an elevated presence of the *Devosia* genus is associated with a higher mortality risk among these patients. Recently, a preclinical study involving experimental rats with adenine-induced CKD and vascular calcification investigated alterations in both the gut and blood microbiomes. Additionally, it explored the correlation between the characteristics of CKD-associated microbiota and variations in kidney function [144]. The findings indicate the presence of *Acinetobacter* in the circulating microbiome is a potential risk factor for chronic kidney disease and its associated vascular complications.

Immunoglobulin A (IgA) nephropathy, also known as Berger’s disease, represents the most prevalent type of glomerulonephritis when compared to other variants of this condition [145]. A study conducted in 2021 examined alterations in the blood microbiome among 20 individuals diagnosed with progressive IgA nephropathy, in contrast to a control group of 20 healthy volunteers [146]. In instances of Berger’s disease, an elevated presence of the class *Coriobacteriia* and species from the genera *Legionella* (Class *Gammaproteobacteria*), *Enhydrobacter* (Class *Gammaproteobacteria*), and *Parabacteroides* was observed in the bloodstream. A significant limitation of the study is the relatively small sample size. Conversely, a notable strength lies in its consideration of alterations in the intestinal microbiome, leading to the conclusion that the blood microbiome does not exert a direct influence on the intestinal microbiome. This finding stands in contrast to the earlier conclusions drawn by Wehedy et al. (2022) [143], which assert a direct correlation between the compositions of the intestinal and blood microbiomes, thereby highlighting the necessity for additional research into other potential mechanisms that may regulate the blood microbiome.

Between September 2021 and January 2023, 90 participants were enrolled in the study, comprising 30 individuals diagnosed with diabetic kidney disease (DKD), 30 with type 2 diabetes mellitus (T2DM), and 30 healthy controls [147]. This research examines biomarkers that can forecast DKD through the analysis of serum metabolites and gut microbiota. In patients with DKD, there was a significant increase in 36 serum metabolites, including alpha-Hydroxyisobutyric acid, accompanying gut dysbiosis with reduced abundances of g_*Prevotella* and g_*Faecalibacterium*. These metabolites exhibited correlations with markers of renal function, specifically the estimated glomerular filtration rate (eGFR) and Urine Albumin-to-Creatinine Ratio (UACR).

A recent investigation analyzed the microbiome composition in the oral cavity, intestines, and bloodstream following kidney transplantation [148]. This study involved a limited cohort of six patients, who were evaluated three months post-transplantation. Preliminary findings indicate that the blood of transplant recipients exhibits a diminished diversity of microbiota, primarily consisting of *Proteobacteria* and *Firmicutes*.

Currently, research on blood dysbiosis in different kidney disorders remains scarce, and there is a notable absence of data regarding the blood microbiome in cases of acute kidney disease.

### 2.6. Changes in Blood Microbiome in Metabolite Disorders

It is known that metabolite levels in fecal and serum samples are altered in individuals with metabolic diseases due to intestinal dysbiosis [149]. Disorders such as obesity, diabetes, and celiac disease (CD) are among the most prevalent and challenging health issues for public health. Beyond lifestyle and genetics, the blood microbiome has emerged as a critical factor influencing these conditions that are usually associated with metabolic dysregulation, inflammation, and the autoimmune response. As mentioned in the previous sections, alterations in blood microbial composition can disrupt immune function, inflammation, and metabolic pathways, linking them closely to disease development and progression. There is an intricate interplay between the microbiota, nutrition, and host genetic and metabolic composition [150].

As a worldwide epidemic, obesity has been defined by metabolic disorders, energy imbalance, an excessive buildup of fat, and persistent low-grade inflammation [151]. The gut microbiota is thought to be one of the possibly causative human–environment interactions for the significant rise in obesity and related mortality and morbidity over the past few decades [152]. It is believed that one of the foundations through which the blood microbiome forms is the gut microbiome. Therefore, alteration in the gut microbiota linked to obesity may potentially have an impact on the blood microbiome [153].

In their study, Chakaroun et al. [154] found a “circulating bacterial signature” that was connected to inflammation and metabolic disorders, indicating that certain bacterial DNA in the blood could be connected to metabolic health. This discovery suggests a possible link between blood microbiota and conditions such as obesity, type 2 diabetes mellitus (T2DM), and CVD [154]. Most of the current research focuses on the relationship between the gut microbiota and obesity, underscoring the need for more research on the blood microbiota’s function in metabolic health.

Asnicar et al. (2021) described relationships between the microbiome and host metabolism and dietary habits in 1098 people with detailed phenotypes [152]. Their study suggests that the blood microbiome is a significant predictor of fasting and postprandial cardiometabolic markers, such as triglycerides and glucose. These findings emphasize the potential of microbiome-based interventions for improving metabolic health. A research project that examined how the blood microbiome of obese patients (*n* = 101) and healthy donors (*n* = 116) was affected by metabolic obesity characteristics, performed metagenomic analysis of the blood microbiome, focusing on sequencing the variable V3-V4 region of the 16S rRNA gene. As a result, it was observed that the blood microbial diversity was higher in patients with metabolically unhealthy obesity (MUHO) than in healthy donors or those with metabolically healthy obesity. Therefore, *Bacteroidetes*, *Firmicutes*, *Proteobacteria*, and *Actinobacteria* were the dominant phyla found in MUHO. The greater frequency of less common phyla such as *Acidobacteria*, TM7, and *Verrucomicrobia* was specifically connected to MUHO, indicating distinctive microbiota changes associated with metabolic health status [153].

In contrast, heightened energy extraction from compounds that would typically be diminished by fecal matter and fat accumulation in individuals with obesity has been associated with elevated concentrations of *Firmicutes* within the gut microbiome. Research indicates that the rise in *Firmicutes* is positively correlated with both the storage of fat and the consumption of digestible energy [151]. Similarly, a shift in the gut microbiota favoring *Firmicutes*, particularly with a high-fat/carb diet, improves energy extraction and causes weight gain, according to Amabebe et al. (2020) [155]. Since this phyla was found abundantly in the blood microbiome of MUHO, it can be stipulated that it may encourage increased fat deposition and calorie absorption, which might lead to obesity.

While the role of gut microbiota in obesity has been extensively studied, there is a paucity of data that explicitly links blood microbial diversity to obesity. The concept of increased microbial diversity, along with the presence of pro-inflammatory taxa such as *Proteobacteria*, is well-documented in research concerning the intestinal microbiome and its association with obesity-related inflammation [156,157]. However, evidence supporting this phenomenon within the blood microbiome remains scarce, despite it being a burgeoning field of investigation.

Diabetes, especially type 2 diabetes, and obesity are closely related due to metabolic and inflammatory processes (especially persistent low-grade inflammation) that are regulated by both the blood and gut microbiomes. Obesity-related excess adipose tissue releases pro-inflammatory cytokines, such as interleukin-6 (IL-6) and tumor necrosis factor-alpha (TNF-α). A major contributing factor to the development of T2DM is insulin resistance, which is caused by these cytokines interfering with insulin signaling pathways. Due to increased intestinal permeability brought on by obesity-associated dysbiosis, pro-inflammatory taxa such as *Proteobacteria* cause microbial translocation and endotoxemia, which in turn cause systemic inflammation [158,159,160]. Insulin signaling is interfered with by this inflammation, which makes glucose intolerance worse. Obesity and weight increase are significant risk factors for insulin resistance and T2DM, and both disorders are exacerbated by chronic inflammation brought on by an excess of lipid buildup. Furthermore, obesity-related changes in microbiota profiles limit the production of healthy SCFAs, which affects the metabolism of fats and carbohydrates (e.g., increased energy extraction) [161,162,163]. Moreover, one of the most researched and prevalent metabolites of gut microbiota are SCFAs, which typically include butyrate, propionate, and acetate [164].

Qiu et al. (2019), in their case-control study, using pre-diagnostic blood samples taken from T2DM patients and controls, directly evaluated the blood microbiome by high-throughput sequencing of the 16S rRNA gene [44]. They then compared the two groups for baseline general blood microbiome composition and relative abundance of particular bacterial taxa. The results show that when compared to non-diabetic controls (non-T2D), the blood microbiome in people with T2D has a greater diversity and distinct microbial patterns. For instance, individuals with the genus *Sediminibacterium* (which is a member of the phylum *Proteobacteria*) are more likely to develop T2DM. On the other hand, the genus *Bacteroides* is inversely related to the development of T2DM. At the same time, T2DM patients have greater abundances of *Actinotalea*, *Alishewanella*, *Sediminibacterium*, and *Pseudoclavibacter* than non-T2D controls, whereas *Aquabacterium*, *Xanthomonas*, and *Pseudonocardia* had lower abundances. Therefore, it can be suggested that the blood microbiome has the potential to be a diagnostic and prognostic tool, and it may also be an etiology factor for developing T2DM [44].

Goraya et al. (2023) observed that blood dysbiosis in T2D patients is typified by a loss of families (*Bacillaceae* and *Bukholderiaceae*) and a decline in orders (*Rhodospirillales* and *Myxococcales*) and genera (*Lactobacillus*, *Acinetobacter*, and *Lactococcus*) in comparison to the healthy population [164]. In a longitudinal investigation conducted by Amar et al. (2011), which included 3280 participants free from diabetes and obesity at the outset and followed them over a period of nine years, the findings indicated that elevated levels of 16S rDNA were associated with the onset of T2DM, independent of other risk factors. Notably, the *Ralstonia* genus emerged as the predominant member of the *Proteobacteria* phylum in the bloodstream of individuals who subsequently developed diabetes [165]. In another study, Sato et al. (2014) found a high rate of gut bacteria in the blood circulation, pointing out that the translocation of bacteria from the gut to the bloodstream is plausible [52]. They found high 16S bacterial rRNA levels in T2DM patients, and *Clostridium coccoides* and the *Atopobidum* cluster were especially frequent. Massier et al. (2020) reported that higher levels of *Thahibacter* are associated with T2DM [166].

Previous studies have largely overlooked the potential influence of the blood microbiome in type 1 diabetes mellitus (T1DM), gestational diabetes, and various other forms of diabetes. The predominant emphasis has been on type 2 diabetes (T2DM) [10,164]. In a recent cross-sectional study, Yuan et al. (2024) examined microbial diversity and its potential relevance to the pathophysiological mechanisms underlying T1DM [167]. This study compared the blood microbiomes of 64 children recently diagnosed with T1DM to those of 77 healthy controls, utilizing 16S rRNA gene amplification and sequencing techniques. The T1D group showed a large rise in the abundance of most phyla, including *Firmicutes*, *Actinobacteriota*, and *Bacteroidota*. Similar to T2DM, *Proteobacteria* was the most abundant phylum in the blood. *Sphingomonas*, *Caulobacter*, and *Stenotrophomonas* were among the potentially harmful species that were significantly more prevalent in the blood microbiome of T1DM patients, and these microorganisms could be involved in systemic inflammation. The blood, gut, and oral microbiomes were shown to be similar, which may indicate the transfer of bacteria carried on by T1DM patients’ impaired oral barrier or increased gut permeability. The authors also emphasized that additional research utilizing transcriptomics, metabolomics, and metagenomics is required to investigate the possible processes underpinning the link between T1DM and the blood microbiome [167]. The connection between T1DM and the dysregulation of circulating microbiota remains to be fully elucidated and explored.

Celiac disease as an immune-mediated enteropathy is triggered by the ingestion of gluten and the associated prolamins (from wheat, barley, and rye) in genetically predisposed individuals [168,169]. This challenging public-health condition is marked by chronic inflammation and malabsorption, small intestinal villous atrophy and crypt hyperplasia in the small bowel [170]. Microbiome changes in the digestive tract have been linked to several autoimmune conditions, and CD is no exception. This is due to the interaction between the immune system and gut microbiota. In individuals with CD, the gut microbiome undergoes significant alterations, reflecting changes in microbial diversity, composition, and functionality. Patients with CD typically display variations in microbial metabolic processes, a reduction in microbial diversity and an imbalance in the relative abundance of different bacterial taxa. These modifications can lead to immunological dysregulation, inflammation, and compromised intestinal barrier integrity, which are key features in the pathogenesis of the disease [171].

The significance of the gut microbiota in CD has been thoroughly studied; however, there are not many studies that particularly address the blood microbiome in this condition. Valitutti et al. (2019) conducted a comprehensive analysis of the relationship between Crohn’s Disease (CD) and changes in gut microbiota in their review, emphasizing the need for additional research to completely grasp these connections, given that no specific microbiological signature has been identified to date [172]. Scher (2016) highlights the intricacy of microbial participation in CD in another study, which examines the microbiome’s function in this condition beyond diet-genetic connections [173].

Since damaged, inflamed, and hence permeable epithelium is the primary pathway via which commensal microorganisms enter the circulation, it would make sense that specific changes should appear in CD bloodstream. Therefore, when analyzing the adult population with CD, Serena et al. (2019) in their research described patients in terms of alterations in the blood microbiome and how these changes may relate to the intestinal microbiome composition and, ultimately, to the loss of tolerance to gluten [174]. They saw that CD patients showed decreased levels of *Clostridiales* (*Firmicutes* phylum) and *Bifidobacterium* and a higher prevalence of *Bacillales* (*Firmicutes* phylum). These modifications align with the alterations in CD-related inflammation and immunological modulation. Moreover, their analysis also revealed that the general microbiome taxonomy of fecal and blood samples differs, with *Firmicutes* and *Bacteroidetes* defining the fecal samples and *Proteobacteria* being abundant in the hematic specimens. Furthermore, the CD blood samples were less phylogenetically diverse compared with controls, with lower alpha diversity. On the other hand, the CD pediatric population was the main subject for Mehrotra et al. (2021) in their case control study [175]. Here, *Bacteroidetes* was the most prevalent phylum, closely followed by *Firmicutes* and *Proteobacteria*. Taxa including *Campylobacterales* (namely *Campylobacter jejuni*, *Campylobacter coli*, *Helicobacteraceae)*, *Odoribacteraceae*, and *Bacteroides acidifaciens*, which have been connected to inflammation and the immune response, were less common in active CD patients. At the same time, it was noted that when compared to controls, those with active CD had higher beta diversity, suggesting notable species compositional changes. Future study is crucial to clarify the blood microbiome’s possible significance in the etiology and development of CD, given there are currently few investigations on this matter. Future treatments that target these microbial changes may be able to supplement the gluten-free diet and provide more effective disease-management techniques.

In the context of metabolic disorders, the blood microbiome represents a significant yet underexplored element. The microbial imbalances identified in the bloodstream, as outlined in Table 4, influence various physiological processes, including immunity, metabolism, and inflammation. Advances in microbiome research and technology have the potential to revolutionize the prevention, diagnosis, and treatment of these disorders, offering hope for improved health outcomes and an enhanced quality of life.

## 3. Discussion

The term “microbiome” can be described as the community of phages, viruses, bacteria, archaea, fungi, and protozoa that occupy a specific ecological niche, including their products and extracellular DNA/RNA [176], i.e., the totality of all microorganisms, genes, and their products in the surrounding environment. In the course of evolution, many microbial species have successfully adapted to the macroorganism. In this regard, it was reported that the human body has 10 times more bacteria than the number of human cells [177].

Blood is a special type of connective tissue, made up of cellular elements and liquid plasma, circulating in a common bloodstream circulatory system. The blood microbiome was until recently unknown and is still poorly studied [26]. It is known that microorganisms do not enter the bloodstream directly. Initially, they are found in the intercellular (interstitial) space of the macroorganism, and then through the lymphatic capillaries they pass into the blood.

Although the blood microbiome is still a mystery, its existence has been conclusively proven over the past 50 years. The assumption of the presence of normal microflora in the blood was made in the last century [178,179,180]. In 1993, the Bulgarian scientist Emil Kalfin expressed the opinion and experimentally proved that normal microflora exists in the blood of healthy people [3]. Kalfin suggested that the blood microbiome, which he called “normal blood flora”, could be activated by stress factors and be cultured.

Numerous studies have provided evidence of multiplying microorganisms in the blood of clinically healthy individuals [3,18,47,181,182,183] or have demonstrated circulating microbial DNA or RNA [5,27,184].

Historically, specific body compartments were thought to be sterile; however, recent evidence has called this belief into question, indicating that different niches, such as blood, contain microbial communities that may affect health. This change signifies an increasing acknowledgment of the microbiome’s significance in human physiology and disease, leading to discussions regarding the consequences of microbial existence in areas previously deemed sterile. Recent studies have explored the concept of a blood microbiome and its potential role in various pathological conditions. Consequently, this review seeks to consolidate the existing knowledge regarding alterations in blood microbiome composition across various disease categories in comparison to healthy individuals.

The emerging evidence from these studies underscores the potential of blood microbiome signatures as diagnostic and prognostic tools in various pathological conditions. Further research is needed to standardize methodologies and validate these findings across larger cohorts.

In this review, we summarized data from studies of the human blood microbiome in disorders of the nervous system, cardiovascular, respiratory, liver, kidney diseases, and metabolite disorders. Links between blood microbiome and the above mentioned diseases were demonstrated. Hence, the human blood microbiome could be considered as a potential biomarker in certain disorders. In support of this understanding, it is evident that analogous alterations in microbiome composition occur across various disease categories; however, the microbial signatures associated with the blood microbiome exhibit specificity. For instance, elevated levels of *Proteobacteria* have been identified in cardiovascular, renal, and metabolic disorders. Conversely, while *Firmicutes* are found to be abundant in renal and metabolic conditions, their levels are diminished in cardiovascular diseases. Additionally, patients suffering from respiratory and liver ailments typically show a heightened presence of *Bacteroidetes*; notably, *Flavobacterium* is prevalent in respiratory diseases, whereas *Enterobacteriaceae* is associated with liver diseases.

It could be assumed that in the next few years, research teams will focus on accumulating new knowledge about the blood microbiome and its relationship to numerous diseases with a suspected infectious etiology, such as some forms of arthritis, sarcoidosis, blood anemia, etc., which are still insufficiently investigated.

## Figures and Tables

**Table 1:** Altered microbiota impact.

Metabolite Production and Absorption	Changes in key metabolites like hippuric acid, indole-3-acetic acid, and glycerol due to altered microbiota
Systemic Inflammation and Metabolic Disorders	Increased gut permeability, systemic inflammation, altered SCFAs, and bile acids
Disease-Specific Metabolomic Changes	Unique metabolomic profiles in chronic hepatitis B and myositis
Gut–Brain Axis	Altered brain signaling molecules and behavior due to dysbiosis
Sex-Specific Responses	Different fecal metabolite changes in males and females post-trauma/sepsis

**Table 2:** Microbiome alterations in CVD patients.

Condition	Increased Compared to Healthy Controls	Decreased Compared to Healthy Controls	Ref.
Individuals to develop CVD	*Proteobacteria*	-	[37]
CVD	*Proteobacteria* (F. *Pseudomonaceae*, Cl. *Gammaproteobacteria*)	*Firmicutes* (F. *Staphylococcaceae*)	[96]
	*Actinobacteria* *Bacteriophages*	*-*	[38]
Hypertension	*Proteobacteria*	*Firmicutes*; *Bacteroidetes*	[100]
Acute coronary syndrome	*Proteobacteria* *Bacteroidota*	*-*	[101]
Chronic coronary syndrome	*Bacteroidota* *Firmicutes*	*Proteobacteria*	[101]
Myocardial infarction	*Actinobacteria*	*-*	[102]
Myocardial infarc-tion	*Armatimonadota* *Caldatribacteriota* *Bacillus* *Pedobacter* *Odoribacter*	*-*	[24]
Vasculitis (Takayasu’s arteritis)	*Clostridia* *Cytophagia* *Deltaproteobacteria*	*Bacilli*	[103]
Vasculitis (giant cell arteritis)	*Cytophagaceae* *Rhodococcus*		[103]
CVD mortality	*Enhydrobacter* *Kocuria*	*Paracoccus*	[39]

**Table 3:** Microbiome alterations in patients with liver damage.

Condition	Increased Compared to Healthy Controls	Decreased Compared to Healthy Controls	Ref.
Alcohol abuse	*Fusobacteria*	*Bacteroidetes*	[119]
Cirrhosis	*Enterobacteriaceae*	*Akkermansia* (Phylum: *Verrucomicrobiota*) *Rikenellaceae* (Phylum: *Bacteroidota*) *Erysipelotrichales* (Phylum: *Bacillota*)	[35]
Cirrhosis with ascites	*Clostridiales* *Cyanobacteria*	*Moraxellaceae*	[33]
Decompensated cirrhosis	*Firmicutes* *Protobacteria* *Bacteroidetes* *Verrucomicrobia*	*-*	[34]
Portal hypertension (cirrhosis)	*Comamonas* (Class: *Betaproteobacteria*) *Cnuella* (Phylum: *Bacteroidota*) *Dialister* (Phylum: *Bacillota*) *Escherichia/Shigella* (Class: *Gammaproteobacteria*) *Prevotella* (Phylum: *Bacteroidota*)	*Bradyrhizobium* (Class: *Alphaproteobacteria*) *Curvibacter* (Class: *Betaproteobacteria*) *Diaphorobacter* (Class: *Betaproteobacteria*) *Pseudarcicella* *Pseudomonas* (Class: *Gammaproteobacteria*)	[120]
Portal hypertension (cirrhosis)—severe symptoms	*Bacteroides* *Escherichia/Shigella* *Prevotella*	*-*	[120]
Acute pancreatitis	*Bacteroidetes* *Firmicutes*	*Actinobacteria*	[117]
Graft rejection after liver transplantation	*Enterobacteriaceae*	-	[123]
Patients who develop liver failure after partial hepatectomy	Diversity		[124]

**Table 4:** Microbiome alterations in metabolic disorders.

Condition	Increased Compared to Healthy Controls	Decreased Compared to Healthy Controls	Ref.
Obesity	*Actinobacteria* *Acidobacteria* *Bacteroidetes* *Firmicutes* *Proteobacteria* *Saccharibacteria (TM7)* *Verrucomicrobia*	-	[153]
Type 1 Diabetes	*Actinobacteriota* *Bacteroidota* *Firmicutes* *Proteobacteria (Sphingomonas*, *Caulobacter*, *Stenotrophomonas)*	-	[167]
Type 2 Diabetes	*Proteobacteria (Sediminibacterium*, *Ralstonia*, *Alishewanella*, *Thahibacter)* *Actinobacteriota (Actinotalea*, *Pseudoclavibacter*, *Atopobidum cl.)* *Firmicutes (Clostridium coccoides)*	*Bacteroidota (Bacteroides)* *Proteobacteria (Aquabacterium*, *Xanthomonas*, *Bukholderiaceae*, *Rhodospirillales*, *Myxococcales*, *Acinetobacter)* *Firmicutes (Bacillaceae*, *Lactobacillus*, *Lactococcus)* *Actinobacteriota (Pseudonocardia)*	[44,52,164,165,166].
Celiac Disease	*Firmicutes (Bacillales)*, *Proteobacteria* *Bacteroidota (Bacteroides)*	*Firmicutes (Clostridiales)* *Actinobacteriota (Bifidobacterium)* *Proteobacteria (Campylobacterales: Campylobacter jejuni*, *Campylobacter coli*, *Helicobacteraceae)* *Bacteroidota (Odoribacteraceae*, *Bacteroides acidifaciens)*	[174,175]

## Abbreviations

The following abbreviations are used in this manuscript:

CD	Celiac disease
CKD	Chronic kidney disease
COPD	Chronic obstructive pulmonary disease
CTP-A	Child-Turcotte-Pugh class A
CTP-B	Child-Turcotte-Pugh class B
CVD	Cardiovascular diseases
DKD	Diabetic kidney disease
DOAJ	Directory of open access journals
eGFR	Estimated glomerular filtration rate
IgA	Immunoglobulin A
IL-6	Interleukin-6
MDPI	Multidisciplinary Digital Publishing Institute
MI	Myocardial infarction
MUHO	Metabolically unhealthy obesity
non-T2D	non-diabetic controls
OCD	Obsessive–compulsive disorder
OTUs	Operational taxonomic units
SCFAs	Short-chain fatty acids
T1DM	Type 1 diabetes mellitus
T2DM	Type 2 diabetes mellitus
TNF-α	Tumor necrosis factor-alpha
UACR	Urine Albumin-to-Creatinine Ratio
